# Reducing Infrared Radiation and Solid Thermal Conductivity by Incorporating Varying Amounts of GnP into Microcellular PMMA

**DOI:** 10.3390/polym17040471

**Published:** 2025-02-11

**Authors:** Antonio Largo-Barrientos, Beatriz Merillas, Ismael Sánchez-Calderón, Miguel Angel Rodríguez-Pérez, Judith Martín-de León

**Affiliations:** 1 Cellular Materials Laboratory (CellMat), Condensed Matter Physics Department, Faculty of Science, University of Valladolid, Campus Miguel Delibes, Paseo de Belén 7, 47011 Valladolid, Spain; antonio.largo.barrientos@uva.es (A.L.-B.); beatriz.merillas@uva.es (B.M.); marrod@uva.es (M.A.R.-P.); 2 BioEcoUVA Research Institute on Bioeconomy, University of Valladolid, 47002 Valladolid, Spain; 3 CellMat Technologies S.L., Calle del Argon 1, 47012 Valladolid, Spain; i.sanchez@cellmattechnologies.com

**Keywords:** microcellular PMMA, graphene nanoplatelets, heat transfer mechanisms, infrared radiation, phonon scattering

## Abstract

Although microcellular foams are potential thermal insulators, their low density and small pore size allow infrared radiation to pass through, increasing the effective thermal conductivity. To address this drawback, graphene nanoplatelets (GnPs) have previously been added to polymethylmethacrylate (PMMA) samples as infrared blockers, enhancing insulation by reducing the radiative component of heat transfer. In this work, the effect of the content of GnPs is studied. Cellular PMMA samples with GnP contents ranging from 0.5 to 10 weight total percentage (wt. %) and pore sizes between 2 and 5 microns were tested. Thermal conductivity measurements showed that GnP additions from 0.5 to 5 wt. % significantly decrease the radiative term, achieving a 33% reduction compared to pure PMMA and reaching thermal conductivity values of 38 mW m^−1^ K^−1^. Moreover, the structural factor is diminished up to 45% in comparison to pure microcellular PMMA, which, in samples with contents of GnPs such as 1 wt. %, results in a reduction in the conductivity of the solid phase. This approach demonstrates that incorporating small contents of GnPs effectively enhances the thermal performance of microcellular foams, a strategy that could be applied to other polymers to achieve better thermal insulation properties.

## 1. Introduction

The overconsumption of fossil fuels has led to a series of environmental and ecological issues in our modern world, including pollution, global warming, and ecological deterioration. To address these challenges, global agreements have been established with the aim of achieving sustainable development. This involves promoting sustainable energy, improving energy efficiency, and reducing greenhouse gas emissions. In fact, buildings energy consumption for heating, cooling, and domestic hot water is the largest energy consumer segment in Europe, contributing to 40% of the European Union’s energy consumption and 36% of its energy-related greenhouse gas emissions [1,2]. One way to face this problem is through the use of effective thermal insulation materials that reduce heat loss in buildings.

Current materials used for building insulation include mineral or rock wool, glass wool, blown cellulose, polystyrene foams, polyurethane foams, spray foams, and phenolic resin foams. The thermal conductivity of these materials is not low enough to fulfill the actual requirements for energy consumption reduction. In addition, these materials should ensure safety for people and the environment. That is, they must have flame-retardant properties, not causing health hazards, being efficient so that large quantities of these materials do not have to be produced and, therefore, being environmentally responsible. Most of the current insulators do not meet these regulations and must be substituted during the following years. For instance, glass wool can cause irritation to the eyes and respiratory tract, while spray foam insulation can emit toxic substances during application, which may persist if not handled properly [3]. Therefore, there is an urgent need to develop new high-quality materials that meet the aforementioned requirements with very low thermal conductivities to reduce heating or air conditioning consumption and contribute to energy efficiency [4].

Among the mentioned materials, cellular polymers stand out as the most promising candidates. Reducing the thermal conductivity of cellular polymers is an interesting strategy for enhancing energy efficiency across various sectors, including construction, packaging, transportation, and aerospace industries [5,6,7,8,9]. Cellular polymers, which are two-phase systems composed of a solid polymer matrix and a dispersed gaseous phase, have drawn significant attention due to their outstanding thermal and sound insulation properties, impressive strength-to-weight ratios, and reduced raw material consumption [10,11,12]. Therefore, cellular polymers serve as highly effective thermal insulators and offer numerous advantages over alternative insulation materials, such as robust compressive strength, efficient sound insulation, ductility, and cost-effectiveness [13,14,15,16]. Furthermore, cellular polymers have the potential to exhibit enhanced or novel properties compared to their original solid structure, making them ideal for diverse applications where efficient insulation and material efficiency are critical [17,18,19].

The previously mentioned properties and, thus, applications of cellular polymers strongly depend on their cell size. Microcellular polymers present cell sizes in the range of a few microns and are interesting materials for thermal insulation [20]. Beyond the advantage of their reduced weight, the presence of minuscule voids imparts excellent impact resistance, thermal insulation, and sorption properties, in particular in comparison to cellular polymers with larger cells [21]. For these reasons, microcellular polymers are promising materials for future thermal insulation.

Heat transfer through cellular materials can be expressed as a sum of four contributions shown in equation 1, in which each term represents a different heat transfer mechanism: convection of gas within the cells ($\lambda_{c}$), conduction through the solid phase ($\lambda_{s}$), conduction through the gaseous phase ($\lambda_{g}$), and radiation through the cell walls and across the cell voids ($\lambda_{r}$) [22].

(1) $$\lambda =\lambda_{c}+\lambda_{s}+\lambda_{g}+\lambda_{r}$$

**Figure 1:**
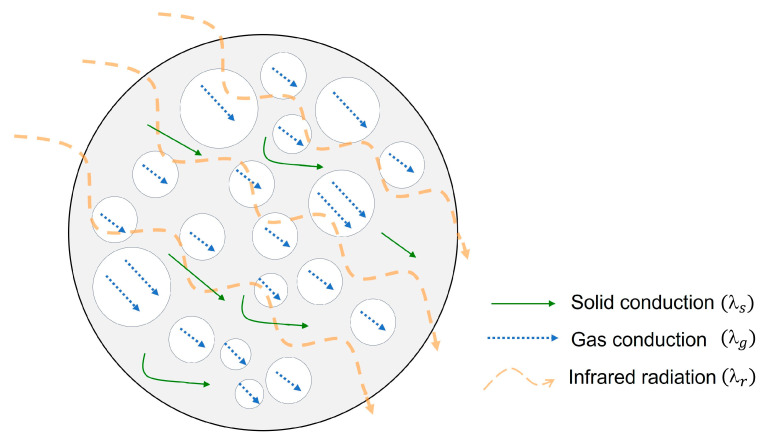
Heat transfer mechanisms of microcellular materials.

The convection of gas within cells can be neglected when the cell sizes are smaller than 2 mm [23]. Since the materials of this work are at least microcellular, this term can be removed from the equation. A schematic representation of the other three heat transfer mechanisms for a cellular material can be found in Figure 1.

On the one hand, conduction through the solid phase ($\lambda_{s}$) is proportional to the thermal conductivity of the polymer matrix ($\lambda_{s}\prime$), an efficiency structural factor named *g* and the relative density (*ρ_s_*) [24]. On the other hand, thermal conduction of the gas phase ($\lambda_{g}$) is related to the heat transfer of the gas inside the cells ($\lambda_{g}^{\prime })$, which is dependent on the temperature and porosity (*v_f_*) of the material, which can be expressed as follows: 1 − ρ_s_ [25]. Finally, the radiative term ($\lambda_{r}$) follows a diffusion-like process governed by absorption of the infrared radiation by the solid matrix and by the scattering on the walls of the cells that are on the cellular structure. This term is proportional to the refractive index of the material (usually considered as 1 for low-density cellular materials), Stefan–Boltzman constant (*σ*), and temperature (*T*) and inversely proportional to the wavelength-averaged extinction coefficient of the material (*Ke*) [26]. These three mechanisms have been analyzed extensively in other studies [24,25,26,27,28,29], so the discussion will focus on the strategies currently used to reduce heat transfer contributions and which other approaches may be effective.

To minimize the gas phase contribution, one direct strategy is to reduce the cell size via the Knudsen effect [30]. However, it has been shown that this reduction results in a higher radiative term [31,32] and increased solid-phase conductivity, primarily due to the elevated structural factor of these materials [33]. For the solid phase, the key lies in minimizing the bulk density. Nevertheless, when the relative density is reduced below 0.2, the solid-phase fraction absorbs a small amount of radiation, leading to an increase in the radiative term [34].

Meanwhile, for reducing the radiative term, current efforts focused on incorporating infrared blockers in micro- and nanocellular polymers have led to improved overall thermal performance. These fillers, which are usually carbon-based, such as graphene nanoplatelets (GnPs) or carbon nanotubes, improve the insulation capacity by two mechanisms: the absorption of thermal radiation and scattering by the matrix, thus reducing the radiative contribution and, therefore, the overall thermal conductivity [35]. There exist several works in which the efficiency of these infrared blockers has been reported. For example, Gong et al. achieved a reduction of 3.5% of the total thermal conductivity of microcellular polystyrene by adding 2 wt. % of multi-walled CNTs [36]. Moreover, in recent research conducted by Martín-de León et al., it is proven that introducing GnPs in micro- and nanocellular polymethylmethacrylate is not only able to reduce the radiative term but also reduces the structural factor *g*, so the total thermal conductivity is lowered in a twofold way. Moreover, the work shows that this method is more effective for microcellular materials than for nanocellular materials as the density is considerably lower for the first ones [27].

Thus, the aim of this work is to find the optimum content of these infrared blockers in microcellular PMMA to achieve the lowest thermal conductivity by effectively reducing heat transfer by radiation. With this purpose, graphene nanoplatelets, which act as infrared radiation opacifiers, are added at varying contents of 0.5–10 wt. % in microcellular PMMA. In addition, it is also analyzed whether the inclusion of these nanoplatelets influences other heat transfer mechanisms and how other key parameters such as relative density, cell size, and nucleation density affect the overall conductivity of cellular PMMA.

## 2. Experimental

### 2.1. Materials

The PMMA PLEXIGLAS 7H used in this work was provided by Evonik Industries in pellet form; this PMMA grade has a density (*ρ*) of 1.19 g/cm^3^ and a glass transition temperature (T_g_) of 112 °C, as specified by the manufacturer. Its melt flow index (MFI) is reported as 0.77 g/10 min, determined under conditions of 230 °C and a load of 2.16 kg.

The grade of graphene nanoplatelets (GnPs) employed is Nano 307 provided by Asbury Graphite Mills. These particles exhibit a laminar structure with a thickness on the nanometer scale (7–8 nm), density of 2.16 g/cm^3^, high aspect ratio, and a lateral size clearly below the micron (0.1 μm).

### 2.2. Sample Preparation

Solid composites were produced through extrusion and compression molding. Firstly, mixtures of PMMA and different contents of GnPs were produced and blended using a twin-screw extruder (model Collin Teach Line ZK 25T). The composites were cooled and pelletized. These mixtures were made from an initial extruded master batch containing 15 wt. % of GnP. In order to fabricate the sheets, they were subjected to compression molding. The extruded materials were dried in a vacuum oven at 80 °C overnight to eliminate any residual moisture. Prior to molding, the materials were preheated in a muffle furnace (model 120/85HA, Nabertherm, Lilienthal, Germany) at 375 °C for 20 min to ensure softening (PMMA reaches a maximum of 300 °C during this process) [27]. Subsequently, the samples were placed on the heated plate of a pressing machine set to 220 °C for 2 min under a pressure of 4.7 MPa. Following this, they were transferred to the machine’s cooling plate, where they were brought to room temperature while maintaining the same pressure. The resulting molded sheets measuring 68 × 68 × 4 mm^3^ were then machined into smaller dimensions of 30 × 30 mm^2^ for the tests.

**Figure 2:**
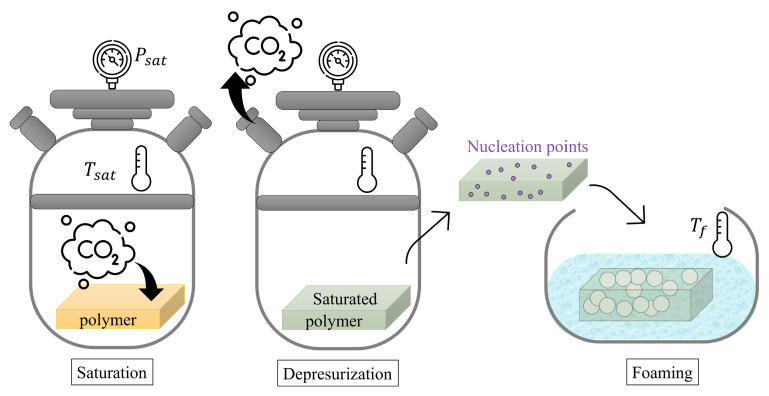
Schematic representation of gas dissolution process.

Later, the cellular materials were produced through gas dissolution foaming with the following conditions: saturation pressure of 50 MPa, saturation temperature of 60 °C, foaming temperature of 90 °C, and foaming time of 90 s. In this methodology, as can be seen in Figure 2, pressurized CO_2_ is dissolved into a polymer matrix under carefully controlled pressure and temperature conditions to promote pore generation upon depressurization. The abrupt pressure reduction creates a state of thermodynamic instability, triggering the formation of small nuclei. These nuclei can then expand into pores when the polymer matrix is heated to temperatures exceeding its effective glass transition temperature.

### 2.3. Characterization

#### 2.3.1. Composition

The filler content in the solid blends was analyzed using a thermogravimetric analyzer (model TGA/SD/TA 861, from Mettler-Toledo, Barcelona, Spain) with a two-step heating program. Initially, samples were heated from 50 to 650 °C under an inert nitrogen atmosphere (N_2_), which causes the PMMA to degrade [37]. In the second step, the temperature was dropped from 650 to 85 °C under air atmosphere, which allowed oxidation of the remaining carbon-based material. Both stages were carried out at a heating rate of 20 °C/min with a gas flow rate of 60 mL/min (either N_2_ or air). The GnP content in the samples was determined by measuring the weight loss during the second heating phase. Three 10 mg samples per material were analyzed to evaluate the consistency of the filler distribution. Additionally, the same procedure was applied to the neat GnP powder, as it could undergo partial decomposition during heating. The filler content for each composite was then adjusted based on the mass loss observed of the GnP particles.

#### 2.3.2. Glass Transition Temperature

The glass transition temperature (T_g_) of the solid composites was evaluated using a differential scanning calorimeter (Mettler DSC30). Samples were heated from 20 to 160 °C at a rate of 10 °C/min under a nitrogen (N_2_) atmosphere, with a gas flow rate of 60 mL/min.

Each sample weighed 5 mg. T_g_ values were determined from the thermograms by identifying the mid-point of the heat flow drop in the first heating cycle. These measurements were influenced by the thermal history of the materials, which result from the extrusion and molding processes they underwent.

#### 2.3.3. Density

The density of solid materials was measured using a gas pycnometer (AccuPyc II 1340, Micromeritics). For cellular samples, the density was determined using the water displacement method, based on the Archimedes principle. This was carried out with a balance (AT261, Mettler-Toledo, Barcelona, Spain) fitted with a density determination kit.

The relative density $\left(\rho_{r}\right)$ is taken as the ratio between the cellular material density $\left(\rho_{f}\right)$ and the density of the solid $\left(\rho_{s}\right)$.

#### 2.3.4. Cellular Structure

Samples were prepared prior to visualization by being cooled in liquid nitrogen and fractured to preserve their original cellular structure. Then, the materials were examined using a compact scanning microscope (model FlexSEM 1000 VP-SEM). In addition, the fractured surface was gold-coated using a sputter coater (SCD 005, Balzers Union).

Following this procedure, SEM micrographs were analyzed using a software based on ImageJ/FIJI [38], obtaining different structural parameters of the materials. Firstly, the average cell size (φ) in 2D was obtained after measuring between 100 and 150 cells per sample. This value is adjusted to 3D after multiplying by a factor of 1.273 [39]. The results were presented together with their standard deviation (SD) to give an idea of the homogeneity of the structure.

Additionally, cell density (*N_v_*) was calculated from images using Kumar’s approximation [40], as defined in Equation (2), which assumes an isotropic distribution of bubbles:

(2) $$N_{v}={\left(\frac{n}{A}\right)}^{3/2}$$

where *A* represents the area being analyzed and *n* denotes the number of cells contained within this area. Subsequently, cell nucleation density (*N*_0_) was calculated using Equation (3), which assumes that no degenerative mechanisms occurred during foaming, with each final cell corresponding to a single nucleation point.

(3) $$N_{0}=\frac{N_{v}}{\rho_{r}}$$

#### 2.3.5. Thermal Conductivity

In order to measure the thermal conductivity of the produced materials, a FOX 200 thermal heat flow meter (TA Instruments, New Castle, DE, USA/Laser Comp Inc., Detroit, MI, USA) was used. The tests followed the steady-state method in accordance with ASTM C518 [41] and ISO 8301 [42] standards. Given that the isothermal plates (300 × 300 mm^2^) were larger than the samples (~60 mm^2^), a methodology validated by Sánchez Calderón et al. [43] was applied. A temperature gradient of 20 °C was maintained between the hot (lower) and cold (upper) plates during each measurement. Measurements were taken at temperatures of 10, 15, 20, 25, and 30 °C, with each experiment lasting a minimum of 30 min to ensure steady-state conditions were met. Heat flux was recorded every second, and an adjacent-averaging process was used to smooth the resulting signal.

In order to determine the specific contribution of each term, a thermal conductivity model developed by Sánchez Calderón et al. was applied [28].

## 3. Results and Discussion

### 3.1. Characterization of the Solid

The characteristics of the compression-molded solid materials employed in this work are summarized in Table 1. The nanoparticle contents used are between 0.5% and 10% of the total weight.

Firstly, the measured amount of filler is slightly above the initial desired value. These measured values are the ones used for the thermal conductivity model. Additionally, a small trend of increasing T_g_ is observed from pure PMMA to materials with nanoplatelets. This can be explained by the fact that the particles contribute to the restriction of the molecular mobility of the polymer chains. However, this increase is generally small. The density of the materials remains almost constant when the graphite nanoplatelets are used, with all samples presenting densities of between 1.18 and 1.20 g/cm^3^.

### 3.2. Characterization of the Cellular Materials

**Figure 3:**
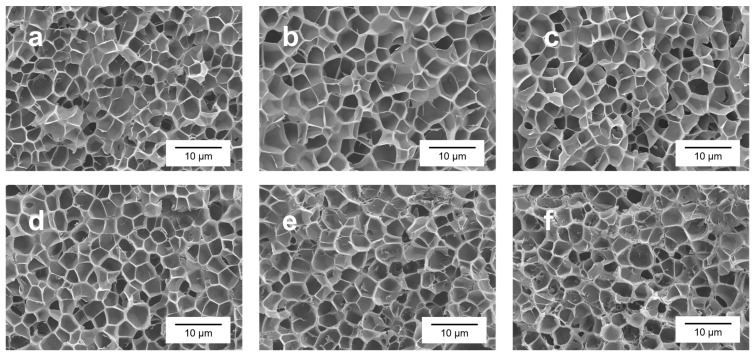
SEM micrographs of the cellular structures of (**a**) pure PMMA, (**b**) 0.5 wt. % content of nanoplatelets (**c**) 1 wt. % content, (**d**) 2 wt. % content, (**e**) 5 wt. % content, and (**f**) 10 wt. % content.

Each sample has been analyzed using SEM (scanning electron microscope), as explained in Section 2.3.4. Some examples of the images obtained are shown in Figure 3. As can be seen in these micrographs, the pure PMMA sample and samples containing GnPs present both a homogeneous microcellular structure; however, the cell size is slightly smaller for the pure material, and seems to be reduced with a growing GnP content. Additionally, cells for pure and small contents of GnPs present a similar aspect with no significative differences (Figure 3a–c). However, for higher contents, the GnPs form aggregates that can be observed from 2 wt. %, but it is especially significant in the 5 and 10 wt. % samples, as can be seen in Figure S2 of the Supporting Information.

These aggregates suggest that the nanoplatelets are not well dispersed through the matrix and, for high quantities, they tend to pile. Counter-intuitively, at first sight, the cell size does not seem to be affected by these aggregates. Macroscopic defects were not taken into consideration for thermal conductivity measurements because their magnitude is similar across all samples, as can be seen in Figure S1 of the Supporting Information.

#### 3.2.1. Cellular Structure and Density

##### Cellular Structure

**Figure 4:**
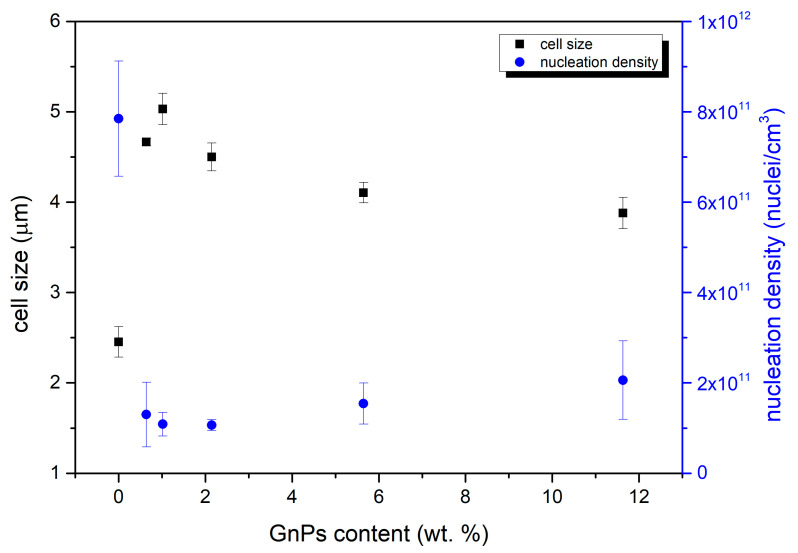
Variation in cell size in micrometers and nucleation density as a function of the total nanoplatelets content.

Cellular parameters were obtained using the Fiji software mentioned in Section 2.3.4. The most relevant ones are the cell size and cell nucleation density represented in Figure 4.

Figure 4 shows how the cell size is higher for all the cellular materials containing infrared blockers in comparison with the reference material. While cell size values are around 2.5 microns for pure PMMA, materials with GnPs present cell sizes in the range of 4 to 5 microns. This cell size slightly increases from 0.5 to 1 wt. % but decreases for the rest of the contents. On the other hand, as expected, the cell nucleation density follows an inverse trend, finding its maximum for the pure microcellular PMMA and the minimum for the sample with a content of 1 wt. %. This can be explained by the fact that samples with a higher nucleation density have more nucleation points, so there are more cells in the same space; therefore, the size of those cells is smaller. Although these soft tendencies exist, it can be concluded that GnPs are not acting as nucleation points in the foaming process, and different contents hardly change the cell size and nucleation density of the materials.

##### Relative Density

**Figure 5:**
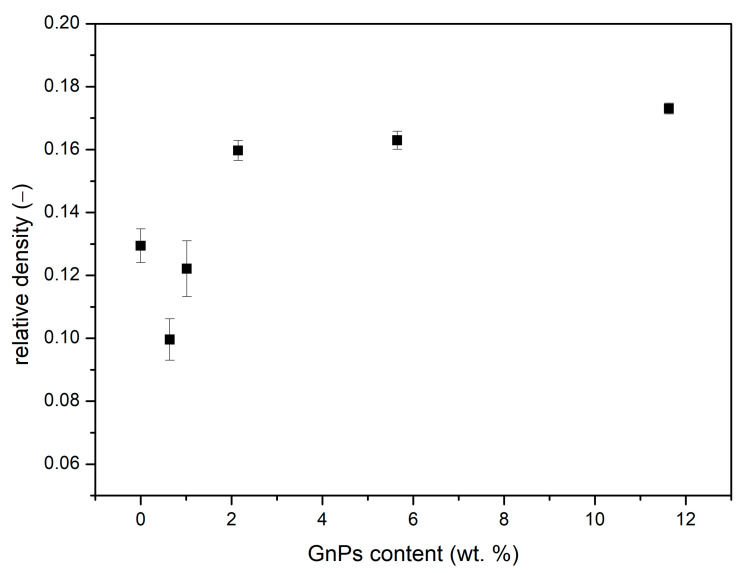
Relative density as a function of GnP content weight total percentage.

As indicated in Figure 5, the relative densities vary in the range of 0.1–0.2. When adding a 0.5 wt. % of GnPs, density is reduced compared to the pure material, from 0.13 to less than 0.10. However, this relative density increases with the content of GnPs up to a maximum value of 0.17 for 10 wt. % of added GnPs. This rising tendency can be explained by the differences in the cell nucleation density and cell sizes, as described in Equation (4) [29]:

(4) $$N_{0}=\frac{6}{\pi \varphi^{3}}\left(\frac{1}{\rho_{r}}-1\right)$$

where $N_{0}$ represents the cell nucleation density, φ is the cell size, and $\rho_{r}$ is the relative density. As stated in Equation (4), the relative density is inversely proportional to the cell size and cell nucleation density. Consequently, as seen in Figure 4, samples with similar nucleation densities exhibit an increment in density as the cell size is reduced. On the other hand, the pure microcellular PMMA sample, despite having the smallest cell size, does not show a significant increase in density due to its high nucleation density.

The relative density has a strong influence on the gas and solid contributions to thermal conductivity, so it is important to take into account the differences in relative density between the samples for the discussion of the results.

### 3.3. Influence of GnP Addition in the Thermal Conductivity

**Figure 6:**
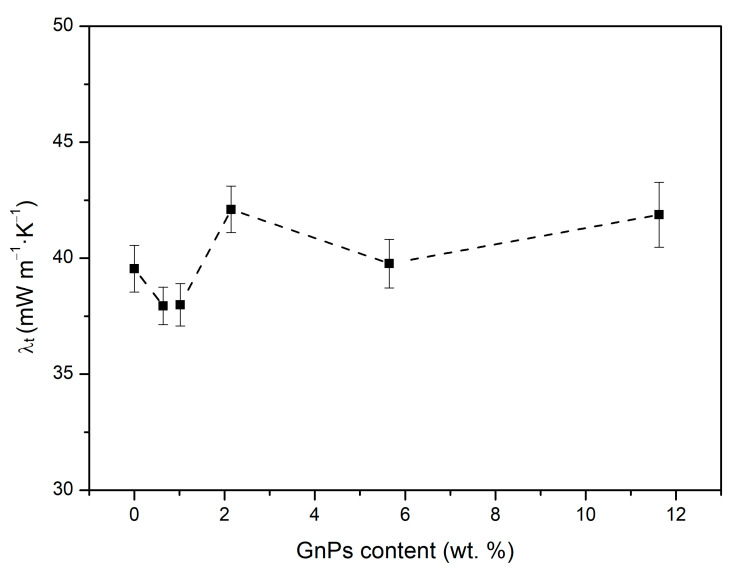
Experimental thermal conductivity measured at 10 °C of PMMA samples with different contents of infrared blockers.

Figure 6 shows the thermal conductivity as a function of GnP content. As displayed, the total thermal conductivity decreases in comparison to the pure PMMA as the nanoparticle content increases up to 1 wt. % content, after which the conductivity increases and even exceeds the values of pure microcellular PMMA. This suggests that several factors must be taken into account in order to understand the origin of these variations.

**Figure 7:**
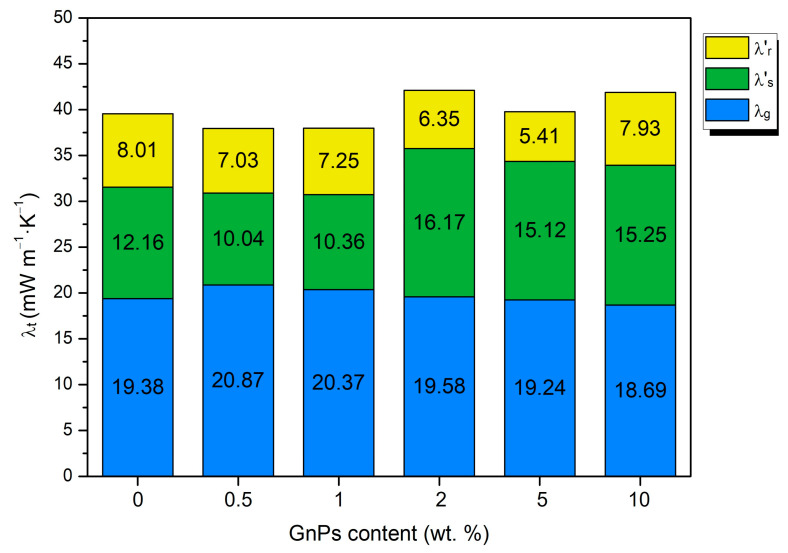
Gas, solid, and radiation contributions to the total thermal conductivity for different contents of infrared opacifiers.

To better comprehend the factors causing these variations, the experimental conductivities have been decomposed into different heat transfer mechanisms. The gas and solid contributions have been calculated theoretically following the Sánchez Calderón method [28], as explained in the Supporting Information (see Figure S3). The contribution of the radiative term has been calculated as the subtraction of the solid and gas contributions to the total conductivity. The different contributions to the total conductivity for each Nano 307 GnP content are shown in Figure 7.

Figure 7 illustrates that the conduction through the gas phase plays a vital role and represents, in all cases, half of the total conductivity (about 20 mW/mK) because of the low relative density of these materials. In addition, the samples present microcellular pores of a substantial size, so the gas phase has considerable importance, unlike in nanocellular polymers. However, differences between samples are small regarding this term.

The second term that mostly contributes to the thermal conductivity is the solid conduction. The solid phase conduction tends to increase for higher contents of nanoparticles, especially from 2 wt. %. This increasing tendency is due, on the one hand, to the increasing relative density with the GnP content (Figure 5) and, on the other hand, to the higher thermal conductivity of GnPs in comparison to PMMA; as the nanoplatelet content increases, $\lambda_{s\;}^{\prime }$ grows as well since both parameters are directly proportional.

Another interesting result that can be inferred from Figure 7 is that the radiative term has been reduced for all GnP contents, obtaining a decreasing trend as the content increases, except for the 10 wt. %, because, as seen in SEM images, for high contents of GnPs, aggregates are formed. In the case of 10 wt. %, these aggregates are so large that they reduce the blocker effectiveness in comparison with the materials with lower contents. The lowest radiative term is achieved for the 5 wt. % content, which represents a reduction of 33% with respect to pure microcellular PMMA. Although the radiative term has been reduced, the inclusion of GnPs makes $\lambda_{s\;}^{\prime }$ higher, which counteracts the effect of the decrease in the radiative term. This, in addition to the bigger relative density values of the 2, 5, and 10 wt. % samples, causes them to have even higher thermal conductivities than pure microcellular PMMA.

**Figure 8:**
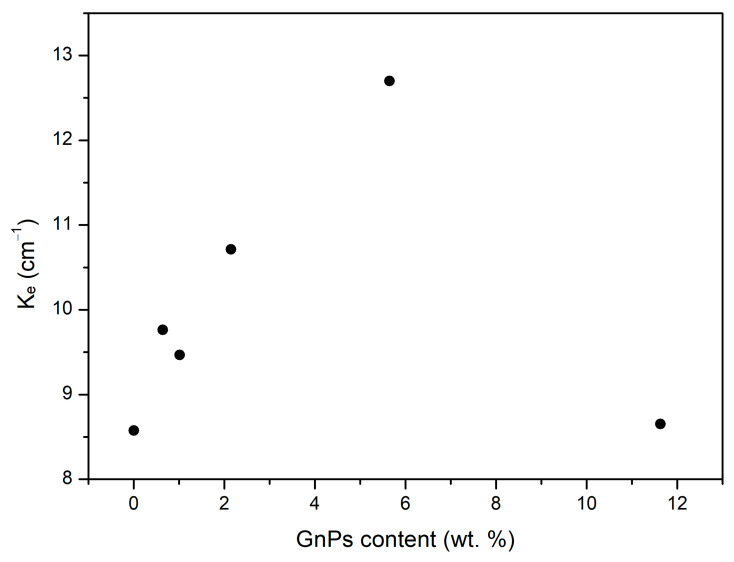
Extinction coefficient for different contents of graphene nanoplatelets.

From the radiative term values, it is possible to calculate the extinction coefficients ${(K}_{e})$. This extinction coefficient is represented in Figure 8, which is inversely proportional to the radiative term, showing again that the added particles efficiently reduce infrared radiation. The highest coefficient is obtained for the sample with the lowest radiative contribution, which is for the 5 wt. % content of graphene nanoplatelets.

After reaching the maximum at a content of 5 wt. %, the extinction coefficient undergoes a drastic decrease for the samples containing 10 wt. % of blockers. This behavior can be attributed to the formation of large aggregates of GnPs in the 10 wt. % samples, as previously discussed. These aggregates hinder the efficient scattering of radiation. Consequently, despite having the highest infrared blocker content, the poor dispersion of the GnPs results in a negligible reduction in the radiative term compared to pure PMMA. This highlights that lower amounts of GnPs are more effective in enhancing performance.

**Figure 9:**
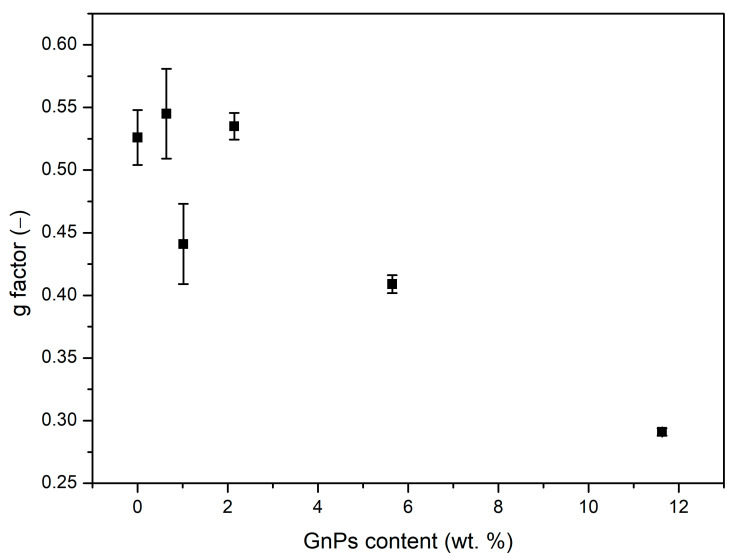
Structural factor *g* obtained by computation.

Although the solid term has been proven to be higher than that of PMMA for high contents of GnPs, it is worth mentioning that samples with 0.5 and 1 wt. % of GnP present smaller values of $\lambda_{s}$ than pure PMMA, even when their density is not smaller. So as to understand this result, the structural factor (*g*) has been calculated by Sánchez Calderón’s method [28]. As illustrated in Figure 9, *g* is diminished compared to pure PMMA, notably for high contents of GnPs. So, although the thermal conductivity of the solid matrix increases due to a high thermal conductivity of GnPs, their presence reduces *g* factor.

This suggests that the conduction in samples with nanoplatelets is reduced in a twofold way through the solid term and the radiative contribution. Scattering of phonons in the polymer–particle interphase could be the explanation for the reduction in the *g* factor, as previously suggested by Martín-de León et al. [29].

Thus, this knowledge is valuable for applying to other particles with lower thermal conductivity to minimize the total thermal conductivity of microcellular PMMA materials.

## 4. Conclusions

Microcellular PMMA with different contents of graphene nanoplatelets (GnPs) between 0.5 and 10 wt. % has been successfully produced and characterized in detail. The SEM images show that the inclusion of these infrared blockers slightly increases the cell size of microcellular PMMA. However, for higher contents, the GnPs tend to form aggregates, although without influence in the cellular structure. In addition, the relative density of the microcellular polymers containing the particles increases when the amount of particles is higher than 2 wt. %.

To analyze the effect of the infrared blockers, the experimental thermal conductivity of each material was measured. A theoretical model was used to determine the different contributions of solid, gas, and radiation terms. The results show a successful reduction in the radiative term with respect to pure microcellular PMMA for every sample with any content of nanoplatelets. The reduction in the radiative term is maximized up to 33% with respect to pure PMMA for a 5 wt. % content of blockers. On the other hand, the content of 0.5 wt. % exhibits the lowest value of total conductivity, reaching 37.94 mW m^−1^ K^−1^. The inclusion of GnPs has also been demonstrated to reduce the structural factor up to 45% in comparison to pure microcellular PMMA, which, in some samples with contents of GnPs such as 1%, is transformed in a reduction in the conductivity of the solid phase, a very interesting fact that could be related to phonon scattering.

Therefore, this study suggests that graphene-nanoplatelet-based infrared blockers can improve thermal insulation in polymeric materials by a dual approach through the solid term and the radiative contribution.

## Figures and Tables

**Table 1:** Main characteristics of the solid materials employed in this study. The table shows the name of the sample, the Nano 307 nanoplatelet filler content as a percentage of the total weight (wt. %), the glass transition temperature (T_g_), and the density of the produced solid precursors (ρ_s_).

Sample	Filler Content (wt. %)	T_g_ (°C)	ρ_s_ (g/cm^3^)
Pure	0	105.73	1.18
0.5%	0.64	107.55	1.19
1%	1.02	108.55	1.19
2%	2.14	108.88	1.20
5%	5.65	110.10	1.20
10%	11.63	109.89	1.18

## Data Availability

The raw data supporting the conclusions of this article will be made available by the authors on request.

## Supplementary Materials

The following supporting information can be downloaded at https://www.mdpi.com/article/10.3390/polym17040471/s1: Figure S1: X-radiography of the different samples. a) Pure PMMA, b) 0.5 wt. % content of GnPs c) 1 wt. % content, d) 2 wt. % content, e) 5 wt. % content, f) 10 wt. % content. Figure S2: Scanning electron micrograph of microcellular PMMA with 5 and 10 wt. % content. Figure S3. Steps followed for the calculation of the solid, gas, and radiative terms contributing to the thermal conductivity.
