# Supplementary material for: Reducing Infrared Radiation and Solid Thermal Conductivity by Incorporating Varying Amounts of GnP into Microcellular PMMA
Source: Polymers (Basel). 2025 Feb 11;17(4):471. doi: 10.3390/polym17040471 (PMC11858911; doi:10.3390/polym17040471)
Supplement: Supplementary file 1 [file polymers-17-00471-s001.zip › polymers-3444803-supplementary.pdf]

Supporting information

Reducing infrared radiation and solid thermal conductivity by incorporating varying amounts of GnP into microcellular PMMA.

A. Largo-Barrientos[1], B. Merillas[1,2], I. Sánchez-Calderón[3], M. A. Rodríguez-Pérez[1,2], J. Martín-de León[1,2]*

[1] Cellular Materials Laboratory (CellMat), Condensed Matter Physics Department, Faculty of Science, University of Valladolid, Campus Miguel Delibes, Paseo de Belén 7, 47011 Valladolid, Spain

[2] BioEcoUVA Research Institute on Bioeconomy, University of Valladolid, Spain

[3] CellMat Technologies S.L., Calle del Argon 1, Valladolid, 47012, Spain

Figure S1 shows radiographs of all the samples. In these images the darker parts correspond to areas of higher density while the lighter parts correspond to areas of lower density. It can be seen that the amount of defects is quite significant for all samples. However, there are not significant differences between the samples so they have not been taken into account for the discussion of the heat transfer mechanisms.

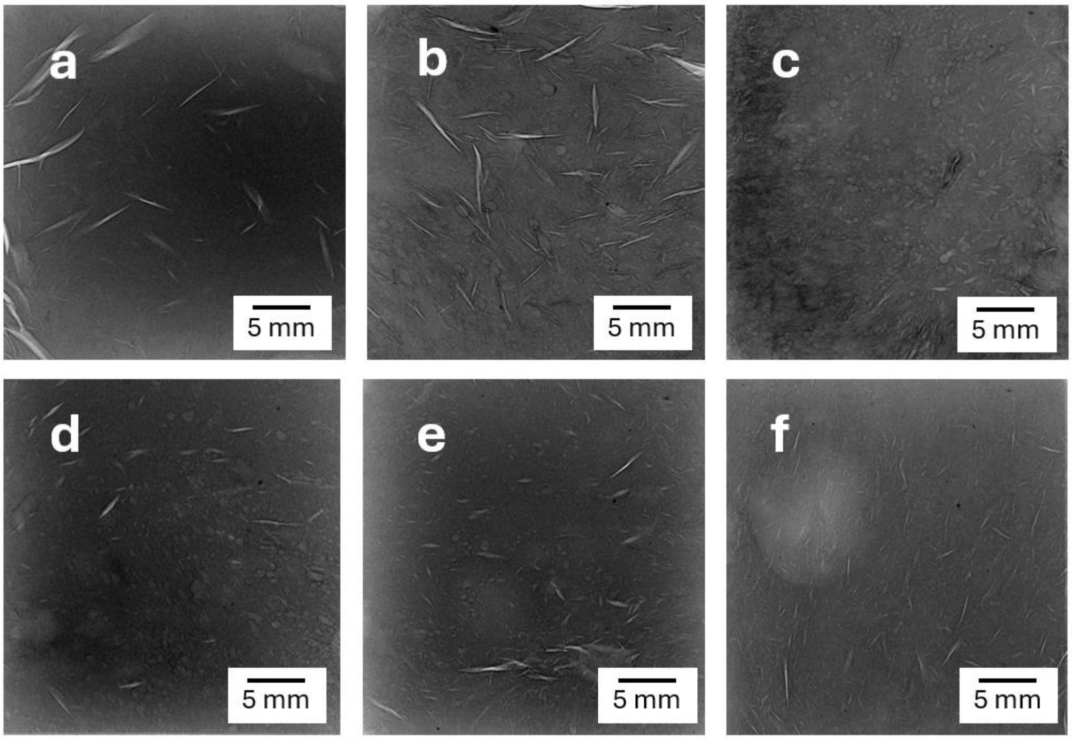

Figure S1. *X-radiography of the different samples. a) Pure PMMA, b) 0.5 wt. % content of GnPs c) 1 wt. % content, d) 2 wt. % content, e) 5 wt. % content, f) 10 wt. % content*

Figure S2 shows zoomed micrographs of samples with 5 and 10 wt. % content. Aggregates of graphene nanoplatelets can be appreciated along the cells.

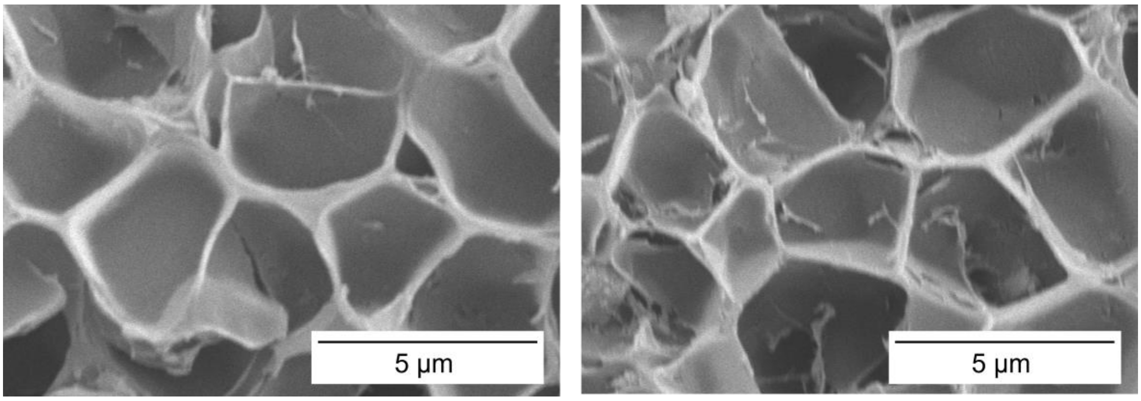

Figure S2. *Scanning electron micrograph of microcellular PMMA with 5 and 10 wt. % content.*

In order to determine the specific contribution of each term a thermal conductivity model proposed by Sánchez Calderón et al. has been used [27]. In Figure S3 a scheme of the procedure followed to obtain the distinct contributions is presented.

Initially, the gas-phase contribution is determined using the equation presented in Step 1, with all parameters known. The value of λ_{air} is extracted from the graph in Step 1 of Figure S2. Subsequently, the solid-phase contribution is calculated using the equation outlined in Step 2, where the structural parameter g is fixed at 2/3. Combined with the relative density, this allows for the determination of λ_s.

Subsequently, the radiative term is easily calculated by subtracting the contribution of the solid and gas term from the total conductivity. Now, as shown in step 4 of Figure 2, the radiative term is plotted against the temperature cubed. As it is known that the radiative term should be 0 mW m^{-1} K^{-1} at 0 Kelvin, the structural factor g is corrected by iteration. In this way, with the corrected g, the slope of the straight line λ_r versus $T^{3,}$ and the equation at step 5, the extinction coefficient K'_e. can be acquired. Finally, a theoretical radiative term (λ'_r) is obtained, which enables to calculate the theoretical solid contribution (λ'_s) as shown in step 6.

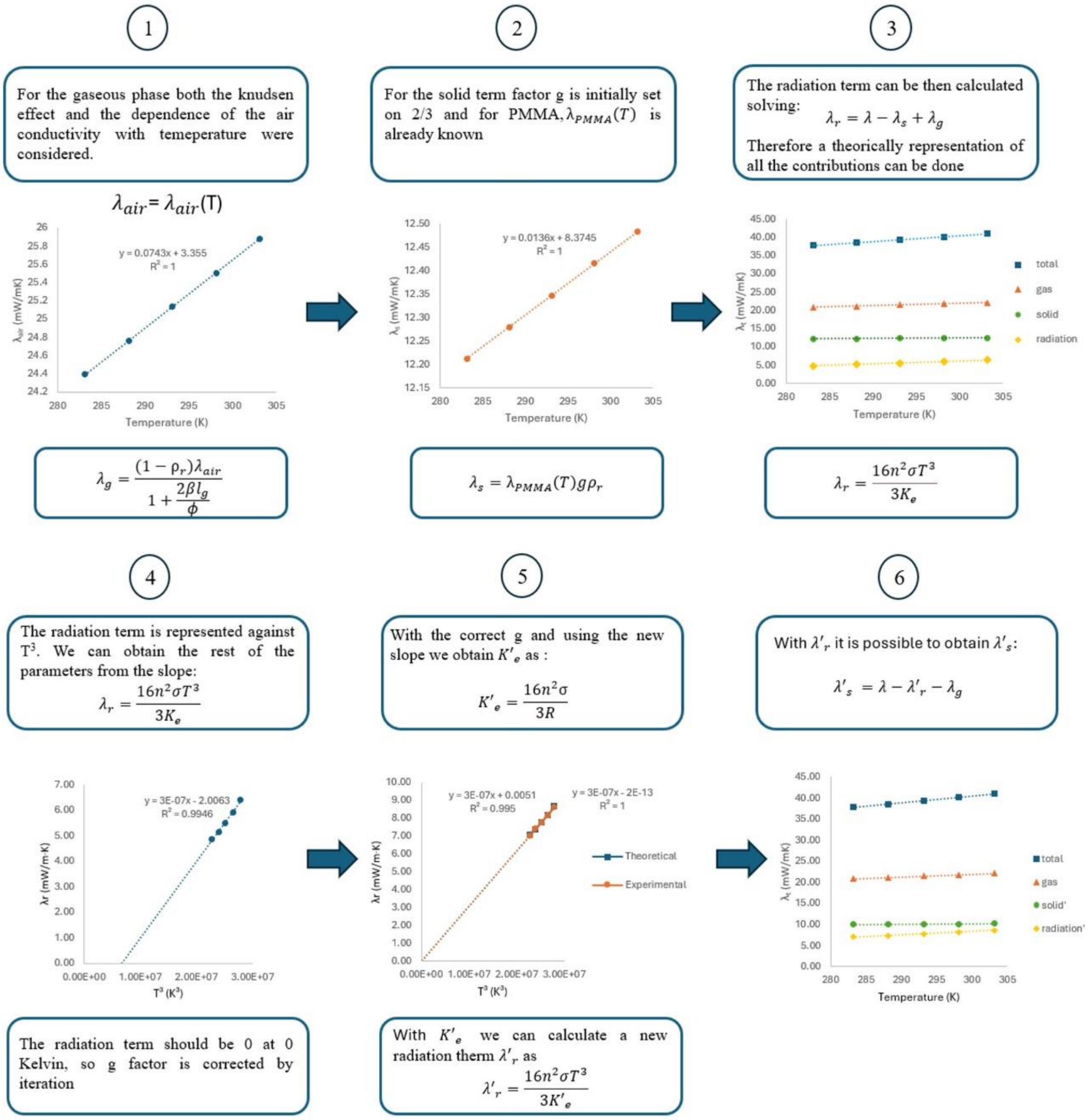

Figure S3. *Steps followed for the calculation of the solid, gas, and radiative terms contributing to the thermal conductivity.*
